# Integrative comparative analyses of metabolite and transcript profiles uncovers complex regulatory network in tomato (*Solanum lycopersicum L*.) fruit undergoing chilling injury

**DOI:** 10.1038/s41598-019-41065-9

**Published:** 2019-03-14

**Authors:** Wen-Fa Zhang, Ze-Hao Gong, Meng-Bo Wu, Helen Chan, Yu-Jin Yuan, Ning Tang, Qiang Zhang, Ming-Jun Miao, Wei Chang, Zhi Li, Zheng-Guo Li, Liang Jin, Wei Deng

**Affiliations:** 1 Key Laboratory of Plant Hormones and Development Regulation of Chongqing, School of Life Sciences, Chongqing University, 401331 Chongqing, China; 2 Department of Plant Sciences, University of California, Davis, CA 95616 USA; 3 Horticulture Research Institute, Sichuan Academy of Agricultural Sciences, Chengdu, 610066 China

## Abstract

Tomato fruit are especially susceptible to chilling injury (CI) when continuously exposed to temperatures below 12 °C. In this study, integrative comparative analyses of transcriptomics and metabolomics data were performed to uncover the regulatory network in CI tomato fruit. Metabolite profiling analysis found that 7 amino acids, 27 organic acids, 16 of sugars and 22 other compounds had a significantly different content while transcriptomics data showed 1735 differentially expressed genes (DEGs) were down-regulated and 1369 were up-regulated in cold-stored fruit. We found that the contents of citrate, cis-aconitate and succinate were increased, which were consistent with the expression of ATP-citrate synthase (*ACS*) and isocitrate dehydrogenase (*IDH*) genes in cold-treated tomato fruit. Cold stress promotes the expression of *ACS* and *IDH* which may increase the synthesis of citrate, cis-aconitate and succinate. Alanine and leucine had increased contents, which may result from alanine aminotransferase (*ALT*) and branched-chain amino acid aminotransferase *(BcAT*)’s high expression levels, respectively. Overall the transcriptomics and metabolomics data in our study explain the molecular mechanisms of the chilling injury and expands our understanding of the complex regulatory mechanisms of a metabolic network in response to chilling injury in tomato fruit.

## Introduction

Low-temperature storage is one of the most effective methods to maintain the nutrients and reduce postharvest decay of fruits and vegetables. However, it is risky to expose tropical and subtropical species to low temperatures, because it can induce the production of a physiological disorder known as chilling injury (CI). CI disorders can cause a reduction in postharvest quality and heavy economic losses^1^.

Tomato, originating from tropical region, is the second-most important vegetable in the world. Tomato fruit are especially susceptible to CI when continuously exposed to temperatures below 12 °C^2^. Chilled tomato fruit symptoms include skin pitting, water-soaking, diseases caused by pathogen, and failure to develop full color^3,4^. CI causes the changes of lipid composition and alterations of conformation and structure in cell membrane, resulting in a decrease of its fluidity and permeability. CI also induces an over-production of reactive oxygen species (ROS) and thus oxidative stress in storage fruit^4^. The ion leakage, malondialdehyde (MDA), proline content, and activities of antioxidative enzymes such as catalase (CAT) and peroxidase (POD) can act as indices to reflect physiological state of plant exposure to CI stress^5^.

Omics-based approaches have been used to understand the complex global biological mechanisms underlying various plant stress responses^6^. A transcriptomic analysis of tomato fruit treated with 6 °C for 48 h found that 38 genes were up-regulated, but only one gene coding a dehydrin was involved in cold-stress genes^7^. An expression analysis of tomato fruit with CI visual symptom after a long period of cold storage (4 weeks at 3 °C) indicated the alterations of genes involved in cell wall modifications, carotenoid biosynthesis, ethylene biosynthesis and signaling^8^. RNA-seq was used to identify differentially expressed genes in tomato fruit after the heat shock, cold treatment and subsequent ripening^9^. The chilling treatment down-regulated the genes related with photosynthesis, metabolism of cell wall, lipid and ethylene, and up-regulated the genes for trehalose synthesis and DOF and MYB transcription factors. On the other hand, the heat shock induced tolerance to CI including the up-regulation of genes involved in heat stress (HSTFs and HSPs), detoxification (GSTs), and sugars metabolism (TPPs and aldose 1-epimerase)^9^.

Proteomic studies in CI tomato fruit revealed that defensive mechanisms were linked to the uncoupling of photosynthetic processes (ATP synthase) and protein degradation machinery (26S proteosome)^10^. Besides, proteomic analysis of cold storage tomato fruit also indicated that the CI tolerance mechanisms were related to the accumulation of heat shock proteins (HSPs), molecular chaperones (GR-RBP), late embryogenesis abundant (LEA) proteins, and antioxidant enzymes (TPxI)^11^. The heat-shock induced chilling tolerance included altering levels of fruit metabolites such as arabinose, fructose-6-phosphate, valine and shikimic acid^12^. Volatile analysis of tomato fruit found that cold storage reduced the production of alcohol, aldehyde, ester, ketone, terpene and acid volatile compounds, and heat shock treatment prior to chilling exposure alleviated the suppression of the key volatile compounds^13^.

Integration of transcriptomic and metabolic profiling data can give important insights into gene-regulatory and metabolic events associated with plant growth and development processes. Recently, a number of studies have begun to integrate the multi-omics data sets, and some of them focused on the development and ripening of tomato fruit^14–16^. In this study, integrative comparative analysis of transcriptomics and metabolomics data from cold treatment tomato fruit was conducted to gain a broader systems perspective and to identify distinct molecular regulatory response during cold storage.

## Results

### Cold storage changes the metabolites profiles in tomato fruit

**Figure 1:**
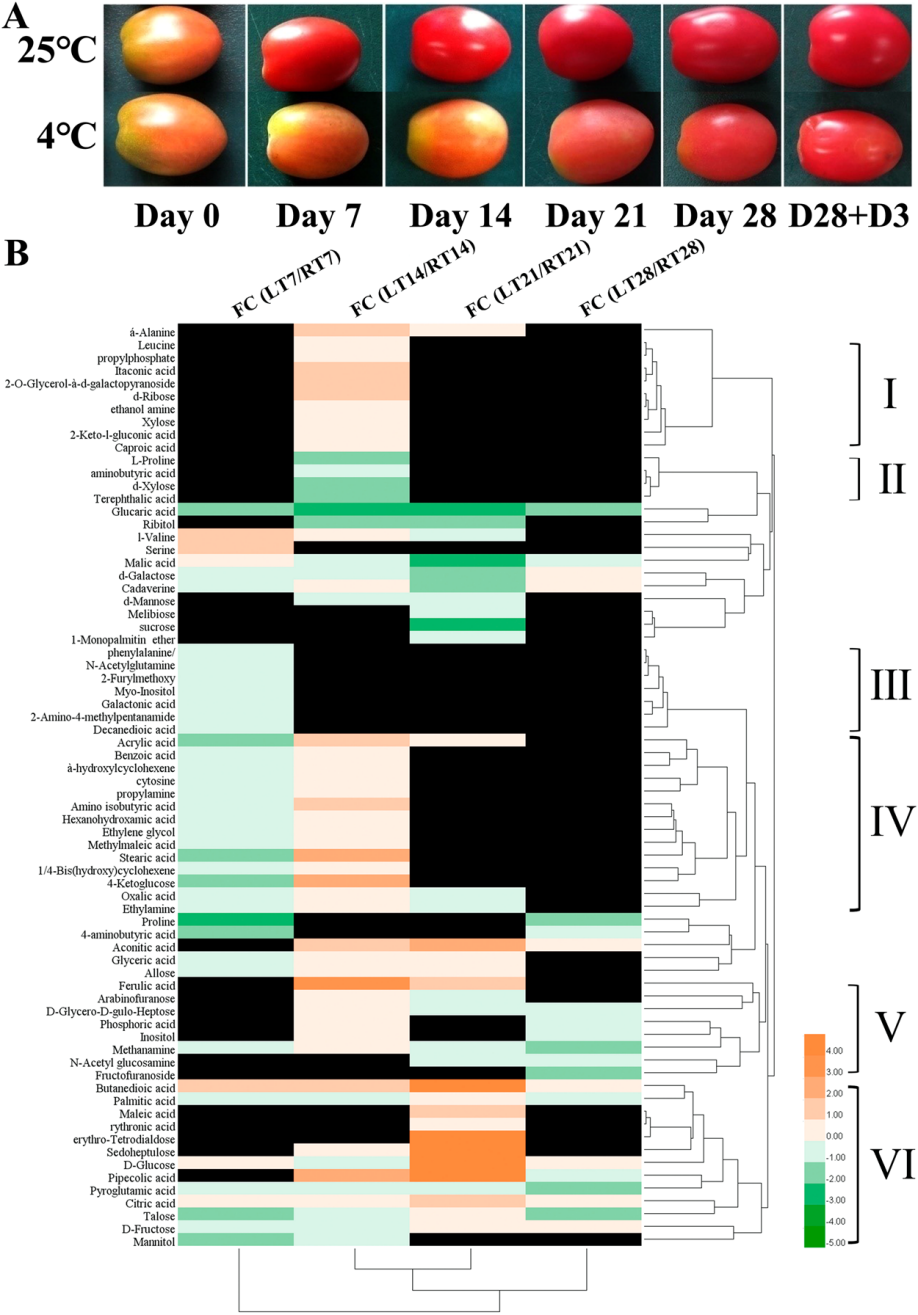
Tomato storage and heatmap analysis of differential metabolites at low temperature (LT) and room temperature (RT). (**A**) Tomatoes were stored for 7 days, 14 days, 21 days, 28 days at 25 °C and 4 °C and then stored at 4 °C to 25 °C for 3 days mimicking shelf storage; (**B**) Fold change (FC) at LT and RT was shown and the blocks colored in red means the up-regulated metabolites and the down-regulated metabolites were colored in green. The blocks colored in black means no significant difference at that time point. Group numbers from I to VI were marked manually by cluster analysis results of the metabolites.

Cherry tomato fruit in the breaker developmental stage were stored at low temperature (4 °C) and room temperature (25 °C) for 28 days. The tomato fruit exhibited typical chilling injury symptoms. Tomato fruit stored at 4 °C (Fig. 1A) delayed the maturation process and 50% of the tomato fruit exhibited skin pitting when storage was continued at 25 °C for 3 days, mimicking the 28 days 4 °C shelf selling storage condition.

To investigate metabolic changes during cold storage, metabolite profiling was analyzed by using gas chromatography–mass spectrometry (GC-MS) method. For the analysis, the tomato fruit were chosen after 0, 7, 14, 21 and 28 days of low temperature treatment. Cold-stored fruit with significantly different metabolites content, including 7 amino acids, 27 organic acids, 16 sugars and 22 other compounds (Fig. 1B, Supplementary Table S1) from each storage stage were selected for further analysis. Cluster analysis showed these metabolites were divided into 6 groups based on their content pattern. In group I metabolites were induced at 14 days of treatment, while in group II the metabolites showed an opposite pattern that was suppressed at 14 days of treatment. The metabolites were inhibited at 7 days in group III. The content was suppressed at 7 days and induced at 14 days of treatment for metabolites in group IV. Most of the metabolites in group V and VI had significantly different contents during long term storage of 14, 21 and 28 days, and the metabolites were significantly induced at 21 days in group VI. It is interesting that the contents of butanedioic acid and citrate were continually induced by cold temperature at 7, 14, 21 and 28 days. However, glucaric acid and pyroglutamic acid showed an opposite pattern of gradually decreasing at 7, 14, 21 and 28 days cold treatment.

### Cold treatment entails extensive transcriptome reprogramming

**Figure 2:**
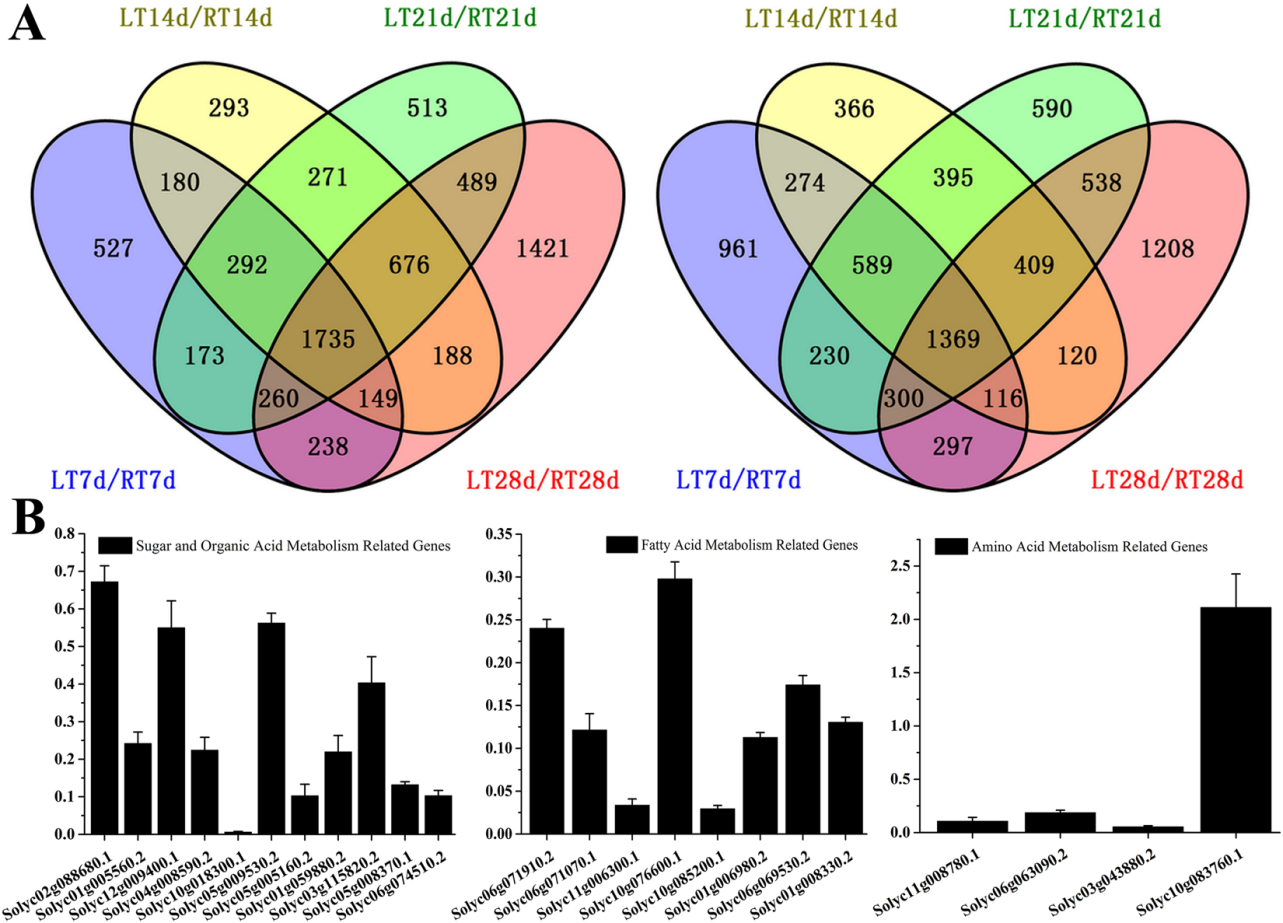
Venn diagram and qRT-PCR validation of DEGs. (**A**) Left figure stands for the down-regulated genes while the right one stands for the up-regulated genes at 4 stages; (**B**) QRT-PCR validation of randomly selected genes related with sugar, organic acid, amino acid and lipid metabolome.

RNA-Seq was performed to obtain a global view of the transcriptome of cold-treated tomato fruit from 7, 14, 21 and 28 days of low temperature treatment. The differentially expressed genes (DEGs) between cold-stored fruit and room temperature stored fruit in each time stage were analyzed to identify the genes that respond to cold stress. A Venn diagram showed that 1735 DEGs were down-regulated and 1369 were up-regulated at each of the five storage stages, which were selected for further analysis (Fig. 2A). These DEGs were useful indicators to identify candidate genes for further in-depth analyses by qRT-PCR. Twenty-two out of 23 genes involved in sugar, amino acids and lipids metabolism, except Solyc02g088680.1 (UDP-glucose 6-dehydrogenase 2), showed similar expression patterns by RNA-Seq assay (Fig. 2B, Supplementary Table S2). The results also indicated that the data from the RNA-Seq were reproducible and reliable.

The DEGs were used for Gene Oncology (GO) and Kyoto Encyclopedia of Genes and Genomes (KEGG) analysis. Interestingly, GO and KEGG analysis indicated a different metabolism network may exist after cold treatment since there were no coinciding GO and KEGG term for down and up-regulated DEGs. GO analysis identified 4 significant terms related with chloroplast and 11 DEGs related with photosynthesis that were up-regulated, which indicated that cold treatment tomato fruit still maintain the function of chloroplast (Table 1, Supplementary Table S3). GO terms was significantly enriched for down-regulated DEGs, integral component of membrane (212 DEGs), plasma membrane (47 DEGs), cell wall organization (14 DEGs), cell wall biogenesis (7 DEGs) as well as plant-type cell wall (13 DEGs) (Table 2, Supplementary Table S4) indicating that cold stress potentially affects membrane and cell wall functioning. Moreover, 24 DEGs involving in calcium binding and 8 DEGs related with calcium-dependent kinase activity were down-regulated, which showed that cold stress decreased the calcium-dependent signal transductions process (Table 2, Supplementary Table S4).

**Table 1:** GO and KEGG enrichment for up-regulated DEGs.

Category	Term	Name	Count	P-Value
Biological Process	GO:0006270	DNA replication initiation	6	0.00207
Biological Process	GO:0052696	flavonoid glucuronidation	16	0.002376
Biological Process	GO:0015979	Photosynthesis	11	0.002461
Biological Process	GO:0009813	flavonoid biosynthetic process	16	0.004199
Biological Process	GO:0009735	response to cytokinin	6	0.005635
Biological Process	GO:0000103	sulfate assimilation	7	0.015144
Biological Process	GO:0006541	glutamine metabolic process	5	0.020492
Biological Process	GO:0009693	ethylene biosynthetic process	4	0.021628
Biological Process	GO:0009773	photosynthetic electron transport in photosystem I	3	0.041851
Cellular Component	GO:0009535	chloroplast thylakoid membrane	33	1.17E-09
Cellular Component	GO:0009570	chloroplast stroma	31	1.45E-07
Cellular Component	GO:0009941	chloroplast envelope	28	1.54E-07
Cellular Component	GO:0009507	Chloroplast	43	9.46E-06
Cellular Component	GO:0009654	photosystem II oxygen evolving complex	7	2.85E-04
Cellular Component	GO:0042555	MCM complex	4	0.001528
Cellular Component	GO:0043231	intracellular membrane-bounded organelle	19	0.003011
Cellular Component	GO:0009579	Thylakoid	6	0.003862
Cellular Component	GO:0019898	extrinsic component of membrane	6	0.004843
Cellular Component	GO:0010319	Stromule	4	0.025165
Cellular Component	GO:0005829	Cytosol	39	0.025596
Molecular Function	GO:0003678	DNA helicase activity	4	0.001548
Molecular Function	GO:0080043	quercetin 3-O-glucosyltransferase activity	13	0.004606
Molecular Function	GO:0080044	quercetin 7-O-glucosyltransferase activity	13	0.004606
Molecular Function	GO:0016671	oxidoreductase activity, acting on a sulfur group of donors, disulfide as acceptor	7	0.013155
Molecular Function	GO:0003824	catalytic activity	14	0.018301
Molecular Function	GO:0030170	pyridoxal phosphate binding	12	0.042889
KEGG_PATHWAY	sly01200	Carbon metabolism	34	5.41E-06
KEGG_PATHWAY	sly00710	Carbon fixation in photosynthetic organisms	16	1.23E-05
KEGG_PATHWAY	sly00195	Photosynthesis	15	1.24E-04
KEGG_PATHWAY	sly01100	Metabolic pathways	137	1.58E-04
KEGG_PATHWAY	sly01130	Biosynthesis of antibiotics	40	0.001247
KEGG_PATHWAY	sly01230	Biosynthesis of amino acids	23	0.006324
KEGG_PATHWAY	sly03030	DNA replication	8	0.012285
KEGG_PATHWAY	sly00030	Pentose phosphate pathway	8	0.018663
KEGG_PATHWAY	sly01110	Biosynthesis of secondary metabolites	76	0.027292
KEGG_PATHWAY	sly00920	Sulfur metabolism	6	0.039347

**Table 2:** GO and KEGG enrichment for down-regulated DEGs.

Category	Term	Name	Count	P-Value
Biological Process	GO:0046777	protein autophosphorylation	9	0.001982
Biological Process	GO:0016132	brassinosteroid biosynthetic process	7	0.003056
Biological Process	GO:0018105	peptidyl-serine phosphorylation	10	0.004176
Biological Process	GO:0071555	cell wall organization	14	0.004704
Biological Process	GO:0042546	cell wall biogenesis	7	0.006139
Biological Process	GO:0010411	xyloglucan metabolic process	7	0.006139
Biological Process	GO:0009738	abscisic acid-activated signaling pathway	10	0.00645
Biological Process	GO:0009408	response to heat	5	0.016273
Biological Process	GO:0035556	intracellular signal transduction	13	0.019121
Biological Process	GO:0032259	Methylation	8	0.023571
Biological Process	GO:0009833	plant-type primary cell wall biogenesis	4	0.028812
Cellular Component	GO:0005802	trans-Golgi network	21	8.92E-08
Cellular Component	GO:0005768	Endosome	17	2.66E-05
Cellular Component	GO:0016021	integral component of membrane	212	0.001422
Cellular Component	GO:0009505	plant-type cell wall	13	0.011292
Cellular Component	GO:0005886	plasma membrane	47	0.012016
Cellular Component	GO:0005794	Golgi apparatus	17	0.021236
Cellular Component	GO:0009506	Plasmodesma	14	0.046915
Molecular Function	GO:0005509	calcium ion binding	24	4.16E-04
Molecular Function	GO:0005516	calmodulin binding	8	0.002049
Molecular Function	GO:0004683	calmodulin-dependent protein kinase activity	8	0.002049
Molecular Function	GO:0009931	calcium-dependent protein serine/threonine kinase activity	8	0.002049
Molecular Function	GO:0005524	ATP binding	96	0.004468
Molecular Function	GO:0016762	xyloglucan:xyloglucosyl transferase activity	7	0.006891
Molecular Function	GO:0016790	thiolester hydrolase activity	3	0.016552
Molecular Function	GO:0008757	S-adenosylmethionine-dependent methyltransferase activity	9	0.033368
Molecular Function	GO:0016759	cellulose synthase activity	4	0.037445
Molecular Function	GO:0016702	oxidoreductase activity, acting on single donors with incorporation of molecular oxygen, incorporation of two atoms of oxygen	5	0.044504
Molecular Function	GO:0003700	transcription factor activity, sequence-specific DNA binding	34	0.047623
KEGG_PATHWAY	sly04626	Plant-pathogen interaction	25	6.73E-06
KEGG_PATHWAY	sly00330	Arginine and proline metabolism	9	0.013333
KEGG_PATHWAY	sly00905	Brassinosteroid biosynthesis	4	0.029545
KEGG_PATHWAY	sly00062	Fatty acid elongation	6	0.031366
KEGG_PATHWAY	sly00520	Amino sugar and nucleotide sugar metabolism	13	0.039295

KEGG annotation suggested that photosynthesis pathways (15 DEGs), the biosynthesis of amino acids (23 DEGs) and the pentose phosphate pathway (8 DEGs) were induced after cold treatment in tomato fruit (Supplementary Fig. S1). On the other hand, fatty acid elongation (6 DEGs), arginine and proline metabolism (9 DEGs) and amino sugar and nucleotide sugar metabolism (13 DEGs) were significantly depressed in cold stress (Supplementary Fig. S1).

**Figure 3:**
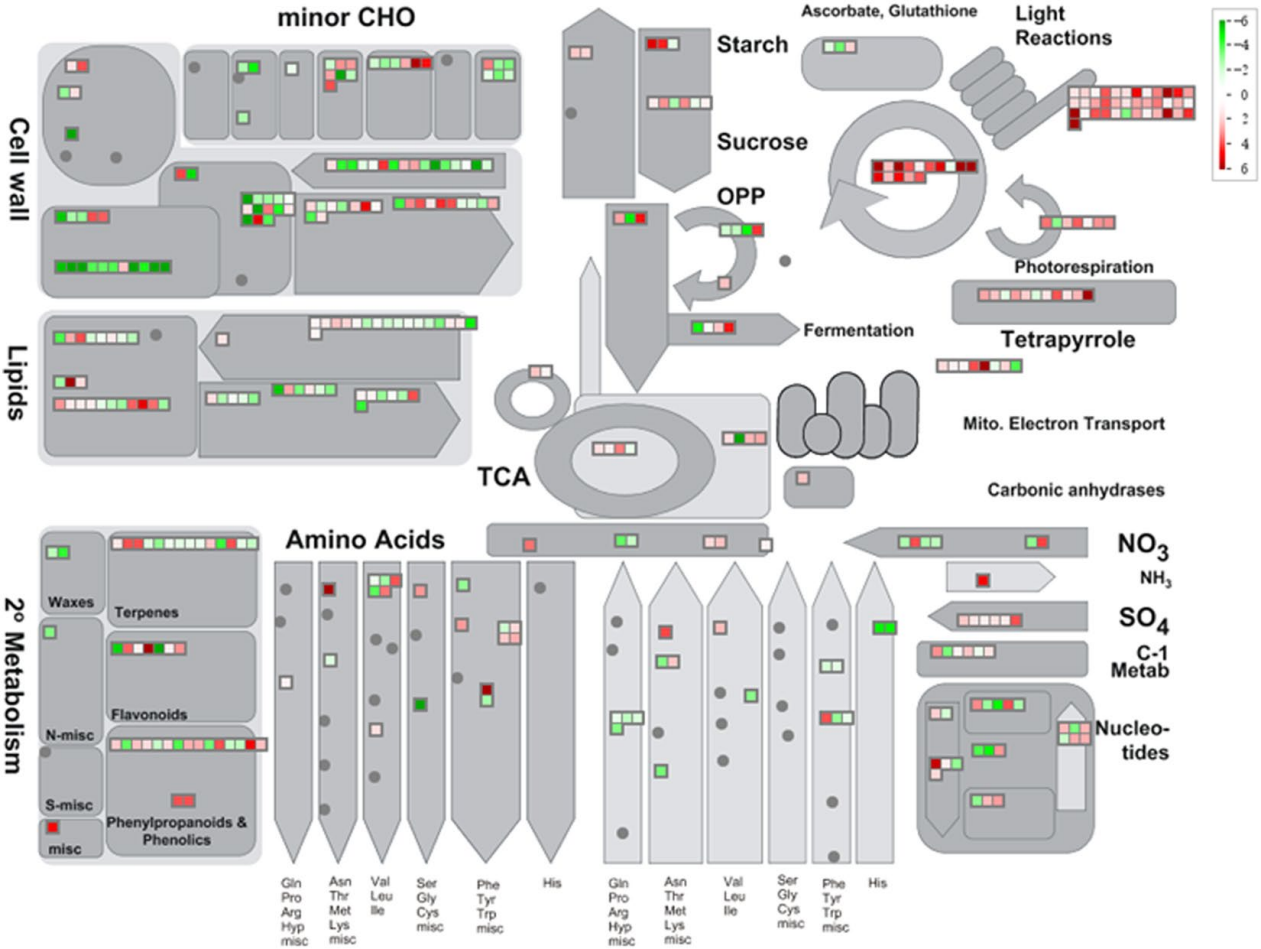
Metabolism network overview of all DEGs by Mapman. The block colored in red and green means the up-regulated genes and down-regulated genes, respectively. The higher the intensity of the color, the greater the difference.

To have a broader view of all DEGs, Mapman analysis was used for the metabolism overview (Fig. 3, Supplementary Table S5). Similar to the GO and KEGG analysis, genes related to cell wall and lipid metabolism have decreased expression levels, whereas genes involved in Calvin cycle and light reactions pathway have increased expression levels after cold treatment in tomato fruit.

### Sugar and organic acid metabolism analysis of cold-stored tomato fruit

**Figure 4:**
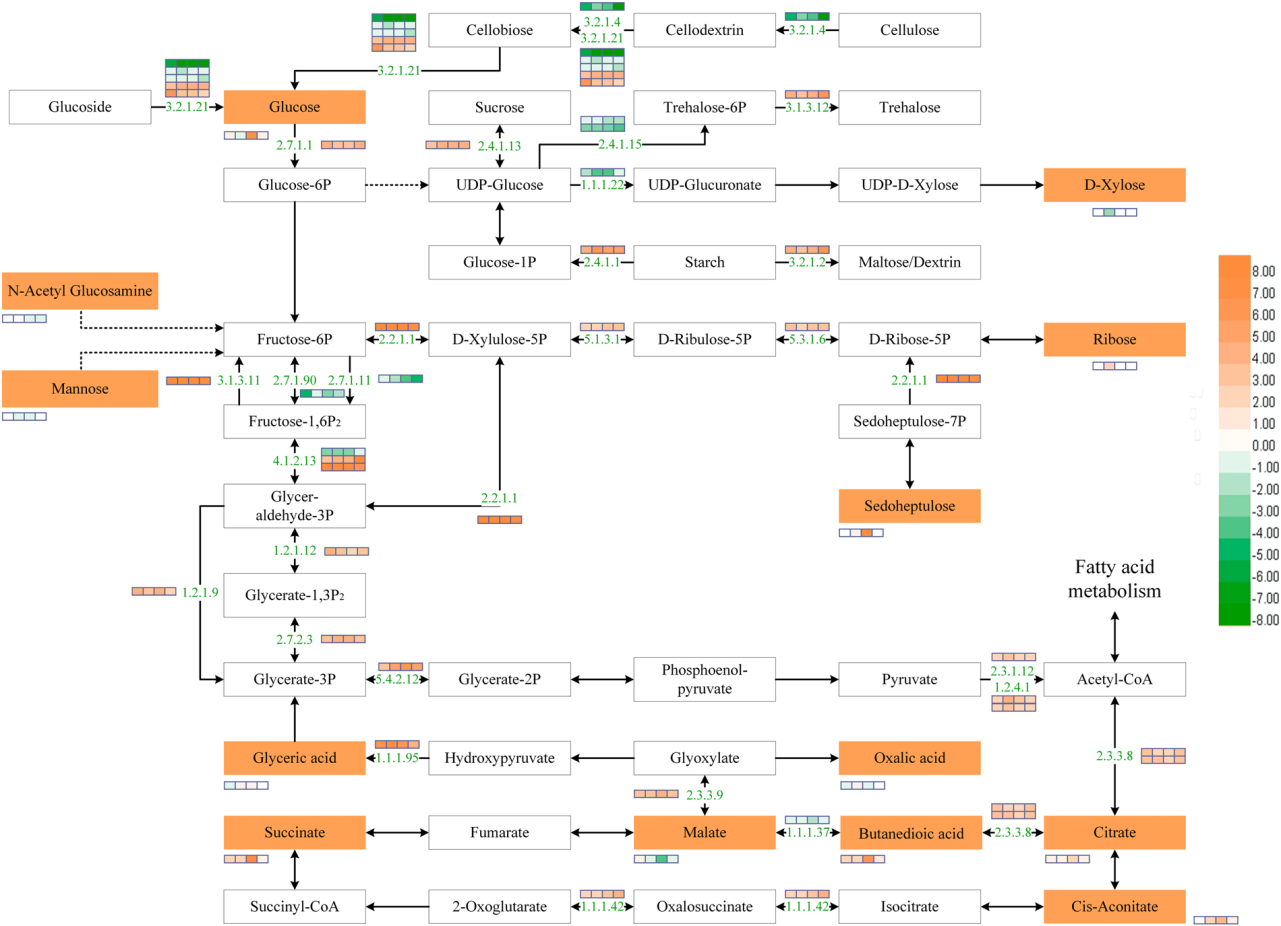
Schematic network of sugar and organic metabolism network. The numbers colored in red are enzyme identifier number. Differential metabolites are in green boxes while the white boxes are other pathway related metabolites. The bars with four small blocks in each DEGs or differential metabolites are the relative fold change value on 7, 14, 21 and 28 days. Orange color means up-regulated under chilling injury and green means down-regulated at that time point. The higher the intensity of the color, the greater the difference. Solid line between two metabolites means a straight reaction while a dashed line means some omission of several metabolites and genes. The enzyme identifier meaning are as follows: 1.1.1.22: UDP-glucose 6-dehydrogenase 2; 1.1.1.37: malate dehydrogenase; 1.1.1.42: isocitrate dehydrogenase; 1.2.1.12: glyceraldehyde-3-phosphate dehydrogenase GAPCP1; 1.2.1.9: NADP-dependent glyceraldehyde-3-phosphate dehydrogenase; 1.2.4.1: pyruvate dehydrogenase E1 component subunit; 2.2.1.1: transketolase; 2.3.1.12: dihydrolipoyllysine-residue acetyltransferase component 4 of pyruvate dehydrogenase complex; 2.3.3.8: ATP-citrate synthase; 2.4.1.1: alpha-1,4 glucan phosphorylase L-2 isozyme; 2.4.1.13: sucrose synthase; 2.4.1.15: trehalose-phosphate synthase; 2.7.1.1: hexokinase; 2.7.1.90: pyrophosphate–fructose 6-phosphate 1-phosphotransferase subunit alpha; 2.7.2.3: phosphoglycerate kinase; 3.1.3.11: fructose-1,6-bisphosphatase; 3.1.3.12: trehalose-phosphate synthase; 3.2.1.2: beta-amylase 3; 3.2.1.21: beta-glucosidase; 3.2.1.4: endoglucanase 24-like; 4.1.2.13: fructose-bisphosphate aldolase 1; 5.1.3.1: ribulose-phosphate 3-epimerase; 5.3.1.6; probable ribose-5-phosphate isomerase 2; 5.4.2.12: probable 2-carboxy-D-arabinitol-1-phosphatase; 2.3.3.9: malate synthase; 1.1.1.95: glycerate dehydrogenase; 2.7.1.11: ATP-dependent 6-phosphofructokinase 6.

Based on transcriptome and metabolome data, a schematic sugar and organic acid metabolism network was built with differential metabolites and DEGs (Fig. 4, Supplementary Table S6). In total, 13 metabolites and 27 DEGs were involved in the network. In the TCA cycle, the contents of citrate, butanedioic acid, cis-aconitate and succinate were increased, which were consistent with the expression of ATP-citrate synthase (*ACS*, EC: 2.3.3.8) and isocitrate dehydrogenase (*IDH*, EC: 1.1.1.42) genes at all four cold treatment stages in tomato fruit. The result indicates cold stress promote the expression of *ACS* and *IDH* genes, thus may increase the synthesis of citrate, butanedioic acid, cis-aconitate and succinate. Besides, malate content showed another pattern: increasing in cold-stored fruit at 7 days and decreasing thereafter. Oxalic acid content was relatively lower in cold-treated fruit at 7 and 21 days storage and higher at 14 days storage.

The glucose content was decreased at 14 days storage and increased at 7, 21 and 28 days storage of cold-treated fruit. The expression level of hexokinase (*HXK1*, EC: 2.7.1.1) was induced at all four cold treatment stages in tomato fruit. Ribose and sedoheptulose had higher contents in cold-treated fruit at 14 and 21 days storage, and transketolase (*TKT*, EC: 2.2.1.1), ribulose-phosphate 3-epimerase (*RPE*, EC: 5.1.3.1), and probable ribose-5-phosphate isomerase 2 (*RPI2*, EC: 5.3.1.6) genes also had higher expression levels in cold-stored fruit. The result demonstrated that cold stress increases the expression of *TKT*, *RPE* and *PRI2* genes, thus may improve the accumulation of ribose and sedoheptulose in tomato fruit.

### Amino acid metabolism analysis of cold-stored tomato fruit

**Figure 5:**
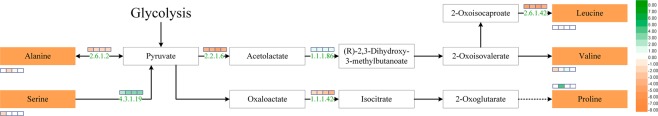
Schematic network of amino acid metabolism. The enzyme identifier meaning are as follows: 2.6.1.2: alanine aminotransferase 2-like; 2.2.1.6: acetolactate synthase small subunit 1; 1.1.1.86: ketol-acid reductoisomerase; 2.6.1.42: branched-chain-amino-acid aminotransferase 5; 4.3.1.19: chloroplast threonine deaminase 1 precursor; 1.1.1.42: isocitrate dehydrogenase.

For amino acid metabolism, 6 DEGs and 5 differential amino acids were involved in the schematic network (Fig. 5, Supplementary Table S6). Alanine and leucine had increased contents in cold-stressed fruit at 14 and 21 days storage, and alanine aminotransferase (*ALT*, EC: 2.6.1.2) and branched-chain amino acid aminotransferase (*BcAT*, EC: 2.6.1.42) had higher expression levels in cold-stored fruit. The higher expression of *ALT* and *BcAT* genes induced by cold stress may improve the synthesis of alanine and leucine in fruit, respectively. The serine content was also increased in cold stressed fruit at 7 days storage, and the expression of threonine ammonia lyase (*TAL*, EC: 4.3.1.19) was decreased in the fruit. The result indicates that cold stress can suppress the expression of *TAL* gene, thus potentially leading to an increase in the accumulation of serine in cold stress fruit. Moreover, proline content was decreased only at 14 days storage and stayed constant at 7, 21 and 28 days storage in cold-stressed fruit.

### Lipid metabolism analysis of cold-stored tomato fruit

**Figure 6:**
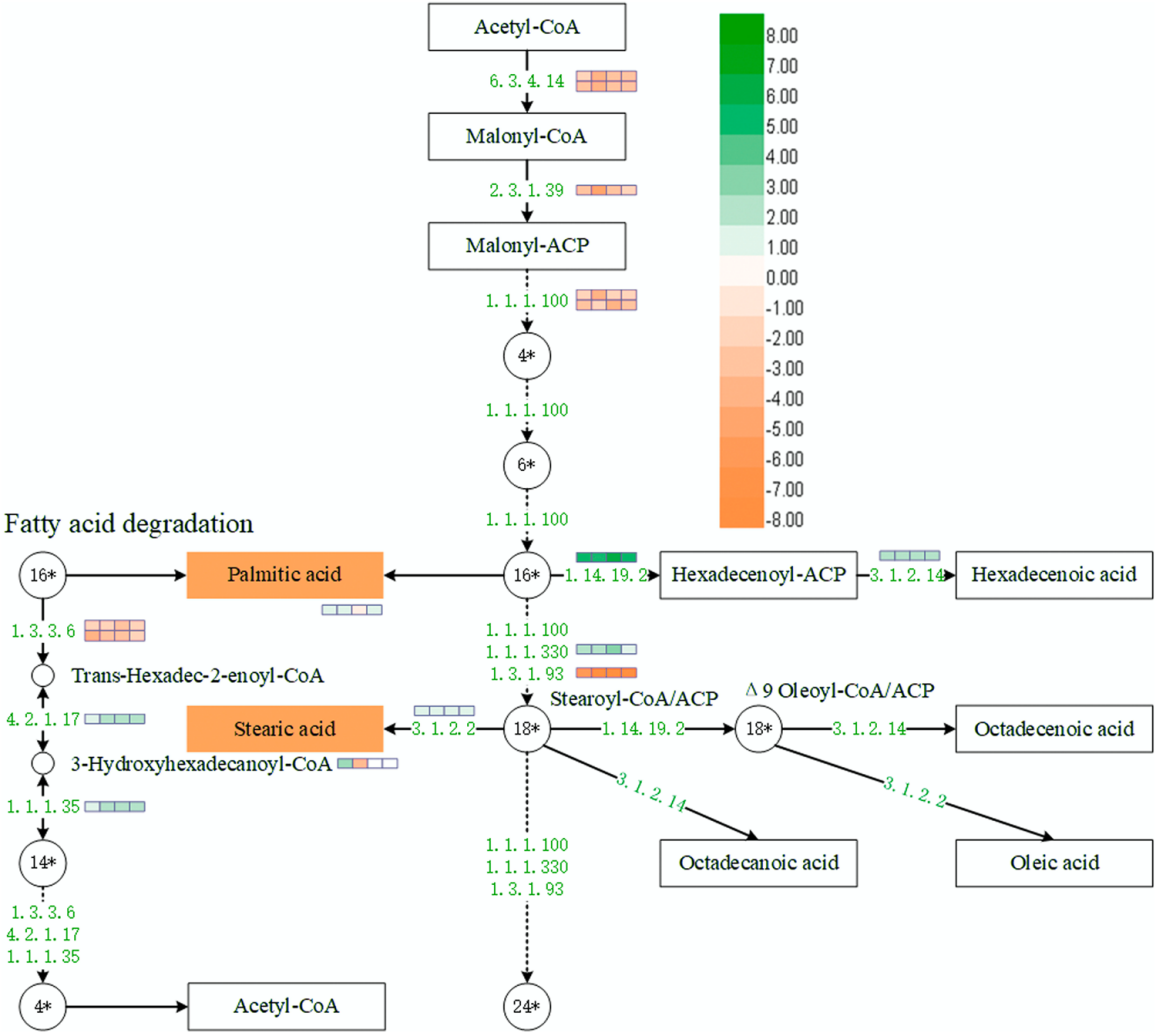
Schematic network of fatty acid metabolism. The enzyme identifier meaning are as follows: 1.3.3.6: acyl-coenzyme A oxidase 4; 3.1.2.22: palmitoyl-protein thioesterase 1; 6.3.4.14: biotin carboxyl carrier protein of acetyl-CoA carboxylase 1; 2.3.1.39: malonyl-CoA-acyl carrier protein transacylase; 1.1.1.100: short-chain type dehydrogenase/reductase-like; 1.1.1.330: very-long-chain 3-oxoacyl-CoA reductase 1; 1.3.1.93: very-long-chain enoyl-CoA reductase-like; 1.14.19.2: acyl-[acyl-carrier-protein] desaturase; 3.1.2.2: acyl-coenzyme A thioesterase 8; 4.2.1.17: peroxisomal fatty acid beta-oxidation multifunctional protein AIM1-like; 1.1.1.35: peroxisomal fatty acid beta-oxidation multifunctional protein AIM1-like; 3.1.2.14: oleoyl-acyl carrier protein thioesterase.

In total, two metabolites and 12 DEGs were involved in fatty acid metabolism pathway (Fig. 6, Supplementary Table S6). The contents of two saturated fatty acids, palmitic acid and stearic acid, were reduced in the cold-stressed fruit after 7 days storage. Intriguingly, stearic acid content increased after 14 days cold storage. Five fatty acids synthesis genes, including two acetyl-CoA carboxylase 1 genes (*ACC1*, EC: 6.3.4.14), one malonyl-CoA-acyl carrier protein transacylase gene (*MCAT*, EC: 2.3.1.39) and two 3-oxoacyl-[acyl-carrier-protein] reductase genes (*OAR*, EC: 1.1.1.100), had increased expression levels in the cold-stressed tomato fruit at all four storage stages. This result indicates that cold stress can increase the synthesis of fatty acids in tomato fruit.

Two other genes involved in the fatty acid elongation process (from 16* to 18*) were successfully mapped^17^. One gene called very-long-chain 3-oxoacyl-CoA reductase 1 (*KCR*, EC: 1.1.1.330) was down-regulated while another gene called very-long-chain enoyl-CoA reductase-like (*ECR*, EC: 1.3.1.93) were up-regulated in tomato fruit at all four cold treatment stages. Moreover, acyl-[acyl-carrier-protein] desaturase gene (*ACPD*, EC: 1.14.19.2) and oleoyl-acyl carrier protein thioesterase gene (*OTE*, EC: 3.1.2.14) were down-regulated at all four cold treatment stages. These results indicated that cold stress may inhibit the transformation of saturated fatty acid to unsaturated fatty acid in tomato fruit.

### Co-expression analysis of DEGs involved in sugar, organic acid, fatty acid and amino acid metabolism

In an attempt to validate whether the DEGs in each metabolism network were “hub genes”, co-expression analysis was performed between the selected genes and other genes involved in the network. Hub genes are genes that have high centralities and play a crucial role in a network.

A total of 57 tomato gene annotations involved in sugar and organic acid, amino acid as well as fatty acid schematic pathway were selected for co-expression analysis (Supplementary Fig. S2). In the sugar and organic acid metabolism network, 7 genes were found to be significantly co-related with *ACS* (EC: 2.3.3.8, Solyc05g005160.2, Solyc01g059880.2) and 1 gene was significantly co-related with *IDH* (EC: 1.1.1.42, Solyc01g005560.2), indicating an important role of ACS in response to cold stress in tomato fruit. In amino acid metabolism, 3 genes were co-related with *ALT* (EC: 2.6.1.2, Solyc06g063090.2) and 1 gene was co-related with *BcAT* (EC: 2.6.1.42, Solyc03g043880.2). In lipids metabolism, the *ACC1* (EC: 6.3.4.14, Solyc01g008330.2), *MCAT* (EC: 2.3.1.39, Solyc01g006980.2) and *FabG* (EC: 1.1.1.100, Solyc06g071910.2) were significantly co-related, indicating a cooperation of fatty acids synthesis in cold-treated tomato fruit.

## Discussion

Tomato fruit are susceptible to CI, resulting in heavy economic losses. With transcriptomic and metabolic conjoint analysis, we were able to reveal some molecular mechanisms of sugar, organic acids, fatty acids, amino acids and other compounds of tomato fruit in response to CI. Even with the caveat that transcript abundance may not lead to changes in enzyme activity, and thus to alterations in metabolite levels, it is nevertheless tempting to speculate that there may be functional. Indications of low temperature increasing the concentration of citric acid are present in other cold-tolerant fruits beside tomato. For instance, banana fruit had the highest concentration of citric acid during the cool dry season harvest^18^. Another example is low temperature induction of the expression of citrate synthase (*CS*) genes and significantly increased citrate content 1.4–1.9 fold in Ponkan fruit^19^. In our results, cold stress increased the content of citrate in tomato fruit. These results indicated that citrate play important roles in response to cold stress in plant fruits and accumulation of citrate may be involved in fruit tolerance to cold stresses.

In the present study, malate content increased in cold-stored fruit at 7 days then decreased. A similar pattern was exhibited in myrtle fruit during cold storage^20^. In contrast, a decline in malate concentration during cold-storage of grape berries has already been reported to exhibit the opposite effect^21^. These examples support our results, indicating the important effect of malate content in cold-stored fruit.

Postharvest treatments with oxalic acid can alleviate chilling injury in sweet cherry by promoting a higher content of bioactive compounds and antioxidant activity^22^. Oxalic acid was also reported to have a CI alleviation function in tomato and mango through exogenous application^23^. In our study, tomato fruit had relatively higher content of oxalic acid at 14 days cold storage. Thus, oxalic acid may benefit the control of CI and the maintenance of fruit quality in cold storage.

The accumulation of organic acids was reported to relate to the plant response to many abiotic stresses. For example, drought treatment induced the accumulation of citrate in the leaves of cotton^24^. Another example is the frost injury increase in malate and titratable acidity in orange fruit^25^. For instance, when plants are under aluminum stress, organic acids, such as citrate, malate and so on are induced and accumulated, resulting in the elimination of aluminum toxicity^26^. These reports suggested that organic acid accumulation may play an important role in plant tolerance to many different abiotic stresses.

Our results indicated that the glucose content was decreased at 14 days storage and increased at 7, 21 and 28 days storage of cold-treated fruit, which resembled a similar pattern found in carambola fruit under cold treatment^27^. Glucose, a central carbohydrate metabolite, was identified to be positively correlated with freezing tolerance^28^. It was reported that a higher content of glucose tends to have a better tolerance of chilling injury in loquat fruit^29^. Cold stress also induced the expression of hexokinase (*HXK1*) gene in tomato fruit. Therefore, the *HXK1* gene may be a response to cold stress and is required for glucose synthesis in tomato fruit under at low temperature.

In this study, alanine, serine, leucine and valine had increased contents in fruit at early cold treatment. Previous studies of peach fruit metabolism affected by cold treatment at 3 days and 5 days confirmed our findings that for alanine, serine and valine contents tended to increase^30^. Obata *et al*. reported that valine and leucine, belonging to branched chain amino acids (BCAAs), were generally accumulated under abiotic stress conditions, including cold stress^31^. BCAAs have a possible role as an alternative electron donor for the mitochondrial electron transport chain under stress. BCAAs can directly provide electrons to the electron transport chain via electron transfer flavoprotein complex, as well as indirectly feed into the Krebs cycle via their catabolic products^31,32^.

Branched-chain aminotransferase (BCAT) enzymes are at the interface of BCAA synthesis and catabolism^33^. Gonda *et al*. reported that the expression of the branched chain amino acid transaminas 2 (Os03g0231600), a BCAT family gene, was induced by both cold and dehydration treatment in rice^34^. In this study, the expression of the *BCAT* gene was induced, and the synthesis of leucine was improved by cold stress in tomato fruit. *BCAT* genes may be a new candidate gene for genetic engineering for cold stress tolerance in plants.

Proline has highly beneficial functions in plants exposed to many stress conditions including chilling injury^35,36^. Proline plays many roles such as being an excellent osmolyte, metal chelator, antioxidative defense molecule and signaling molecule during stress^37^. However, our study showed the proline content was decreased at 14 days storage of cold-treated fruit. We suggested that proline may be induced by cold stress only at the first several days in tomato fruit.

Low temperature storage at 4 °C can restrain the maturation process of tomatoes from breaker stage (Fig. 1A). However, 50% of tomatoes had skin pitting and electrolyte leakage and it was present only after transferring to room temperature for 3 days. Sharom *et al*. observed similar symptoms in tomatoes transferred from 5 °C to 25 °C, possibly due to the lipid phase change in membrane^38^.

CI has an effect on membrane permeability, including electrolyte leakage, lipid phase transitions and changes in lipid composition^39–42^. Our GO analysis confirmed that CI regulate genes significantly enriched in membrane related terms, including up-regulated extrinsic component of membrane, intracellular membrane-bounded organelle and down-regulated integral component of membrane, and plasma membrane. Moreover, cell wall metabolism has been found to play important roles in CI in fruits^43–45^. Recently, an integrative analysis reported that CI significantly regulates long non-coding RNAs targeting cell wall degradation in tomato fruit^46^. We found that cell wall related terms like cell wall organization, cell wall biogenesis as well as plant-type cell wall was down-regulated, indicating CI may induce cell wall degradation and thus may easily lead to skin pitting and electrolyte leakage at shelf storage.

It was also reported that a higher proportion of unsaturated fatty acids produced higher tolerance to low temperature stress. Examples are found in banana, pomegranate and loquat fruit^47–49^. Membrane lipids from chilling-resistant plant species exhibited higher content of unsaturated fatty acids than sensitive species^50–52^. For instance, the decrease in lipid unsaturation was related to the induction of CI in loquat fruit^47^. It was also reported that a decrease in unsaturation of peel tissue occurred during the early phase of CI in chilled cucumber fruit^53,54^. In the present study, acyl-[acyl-carrier-protein] desaturase gene (EC: 1.14.19.2) and oleoyl-acyl carrier protein thioesterase gene (EC: 3.1.2.14) was down-regulated at all four cold treatment stages. The results indicated that cold stress may inhibit the transformation of saturated fatty acid to unsaturated fatty acid in tomato fruit, thus decreasing the contents of unsaturated fatty acids in chilled tomato fruit.

## Conclusions

Our results indicate that cold stress promotes the expression of *ACS* and *IDH* genes, which may have increased the synthesis of citrate, cis-aconitate and succinate. The improved expression of the *ALT* and *BcAT* genes induced by cold stress may improve the synthesis of alanine and leucine in fruit, respectively. In this study, integrative comparative analysis of transcriptomics and metabolomics data uncovered complex regulatory network in tomato fruit under chilling injury. The DEGs found can act as the indicator genes to reflect the physiological state of plants exposed to CI stress. The DEGs can also act as candidate genes for genetic engineering to improve the fruits tolerance to chilling in further studies.

## Materials and Methods

### Plant material and experimental treatment

Cherry tomato (*Solanum lycopersicum* L. cv. Micro-Tom) was harvested from Baishiyi orchard, Chongqing, China. Ninety tomato fruit with the same developmental stage (breaker) were carefully selected for storage experiment at low temperature (4 °C) and room temperature (25 °C), separately (Fig. 1A). Tomato fruit were stored for 28 days and around 5 tomatoes from each group were collected every 7 days and stored in a −80 °C freezer for metabolome and transcriptomic analysis.

### Gas chromatography–mass spectrometry (GC-MS) metabolome analysis

Six biological replicates were used for each group at each time stage in the GC-MS experiment. Tomato fruit were ground into mixed powder and for each replicate a 400 mg sample was collected and mixed with 2 ml of methanol then vortexed for 10 s before ultrasonic extraction at 70 °C. The supernatant was collected after centrifugation at 12,000 rpm. Two milliliter of water and 1 ml of chloroform was added to the test bottle and centrifuged at 14,000 rpm for 5 minutes. A 400 µl sample of supernatant was collected into the test bottle and dried in nitrogen. Later, 80 µl of a 15 mg/ml methoxyamine pyridine stock solution was added to the pellet and vortexed for 30 s. The reaction was incubated at 37 °C for 90 min. Finally, add 80 µl BSTFA with 1% TMCS to the reaction and incubate at 70 °C for 60 min for GC-MS analysis.

GC-MS analysis was performed on an Agilent 6980 GC system equipped with a 30.0 m × 0.25 mm i.d. fused-silica capillary column with 0.25 μm HP-5MS stationary phase (Agilent, Shanghai, China). The inject temperature was set at 250 °C. The column temperature was initially kept at 80 °C for 3 min and then increased from 80 °C to 280 °C at 10 °C/min, where it was held for 2 min. Helium was used as the carrier gas at a constant flow rate of 1 ml/min through the column. A split-less injection volume of 1 µl was used. The column effluent was introduced into the ion source of an Agilent 5973 mass selective detector (Agilent Technologies). The MS quadrupole temperature was set at 150 °C and the ion source temperature was 230 °C. Masses were acquired from m/z 50 to 800. The mass accuracy of the instrument was 0.1 atomic mass unit (amu). The acceleration voltage was turned on after a solvent delay of 180 s.

### Transcriptomic analysis

QIAGEN RNeasy Plant mini kit (Qiagen, Hilden, Germany) was used for RNA extraction of tomato fruit. RNA-seq was performed in Shanghai Majorbio Biopharm Technology Co., Ltd described by Zhang^55^. RNA-seq was performed without replications, acting as indicators only to identify candidate genes which need further analyses by qRT-PCR. Strict criteria were adapted to ensure accuracy. Based on the distribution of the raw data, all sequences were of high quality. All raw data were further filtered to obtain high quality data (Supplementary Fig. S3). All clean reads were aligned to the tomato genome (ITAG 2.3, http://solgenomics.net/) using Tophat (http://tophat.cbcb.umd.edu/). To identify differentially expressed genes (DEGs), transcript abundance was normalized by FPKM (Fragments per Kilobase of exon model per Million mapped reads)^56^ method using R (https://www.r-project.org/). A FDR < 0.05 and |logFC| > 1 was used to determine the threshold for DEGs^57^.

### Gene Oncology (GO) and KEGG function enrichment analysis

GO and KEGG function enrichment analysis was carried out through DAVID bioinformatics resources 6.8 (https://david.ncifcrf.gov/home.jsp)^58,59^. All tomato ID was converted to Entrez ID through Gene Accession Conversion Tool of DAVID before function enrichment analysis. Ease score, a modified Fisher Exact *P*-value, was used for gene-enrichment analysis. Ease score <0.05 was considered strongly enriched in the annotation categories and was accepted for this analysis.

### Mapman network analysis

Mapman (http://mapman.gabipd.org/, version 3.6.0) was used to provide a graphical overview of tomato metabolic networks^60–63^. It is a user-driven tool which can display large datasets onto diagrams of metabolic pathways or biological processes. A table with tomato gene ID and log transformed data (RT/LT) was imported and visualized.

### Co-expression analysis

Gene expression data of 4 storage stages (7, 14, 21 and 28 days) were selected and imported to Cytoscape (http://cytoscape.org/, version 3.4.0) for co-expression analysis. Pearson’s correlation was performed and *P*-value < 0.05 was considered significant.

### Quantitative reverse transcription PCR (qRT-PCR) expression analysis

Total RNA was extracted using RNAprep Pure Plant Kit (Tiangen). First strand cDNA was reversed transcribed from 1 μg total RNA using the First Strand cDNA Synthesis Kit (Thermo Scientific). qRT-PCR was carried out as described previously^55^. The primers used are listed in Supplementary Table S2.

### Ethics approval and consent to participate

The experimental research on plants carried out in this work complies with institutional, national, and international guidelines.

## Supplementary information

Supplementary Figures

Supplementary Table S1

Supplementary Table S2

Supplementary Table S3

Supplementary Table S4

Supplementary Table S5

Supplementary Table S6

## Data Availability

All data generated or analyzed during this study are included in this published article and its supplementary information files.
