# Supplementary material for: Integrative comparative analyses of metabolite and transcript profiles uncovers complex regulatory network in tomato (Solanum lycopersicum L.) fruit undergoing chilling injury
Source: Sci Rep. 2019 Mar 14;9:4470. doi: 10.1038/s41598-019-41065-9 (PMC6418210; doi:10.1038/s41598-019-41065-9)
Supplement: Supplementary file 1 — Supplementary Figures [file 41598_2019_41065_MOESM1_ESM.pdf]

Title:

Integrative comparative analyses of metabolite and transcript profiles uncovers complex regulatory network in tomato (*Solanum lycopersicum L.*) fruit undergoing chilling injury

Authors:

Wen-Fa Zhang[1], Ze-Hao Gong[1], Meng-Bo Wu[1], Helen Chan[2], Yu-Jin Yuan[1], Ning Tang[1], Qiang Zhang[1], Ming-Jun Miao[3], Wei Chang[3], Zhi Li[3], Zheng-Guo Li[1], Liang Jin[1]*, Wei Deng[1]*

[1]Key Laboratory of Plant Hormones and Development Regulation of Chongqing, School of Life Sciences, Chongqing University, 401331 Chongqing, China. [2]Department of Plant Sciences, University of California, Davis, CA 95616. [3]Horticulture Research Institute, Sichuan Academy of Agricultural Sciences, Chengdu 610066, China.

E-mail address of each author:

Wenfa Zhang: fasq2010@163.com; Zehao Gong: 824601591@qq.com; Mengbo Wu: 664313622@qq.com; Helen Chan: hmchan02@yahoo.com; Yujin Yuan: 737851915@qq.com; Ning Tang: 154978305@qq.com; Qiang Zhang: 303113412@qq.com; Mingjun Miao: 308582144@qq.com; Wei Chang: 471243498@qq.com; Zhi Li: 372435194@qq.com; Zhengguo Li: zhengguoli@cqu.edu.cn; Liang Jin: jinliang4002@126.com; *Correspondence to Wei Deng: dengwei1977@cqu.edu.cn.

Supplementary Fig. S1:

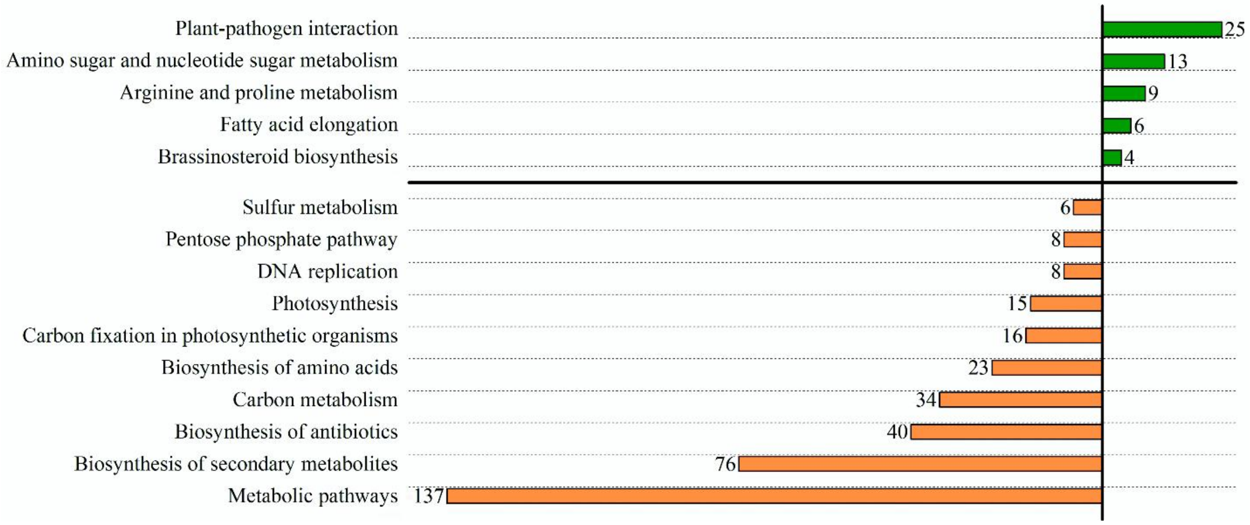

Supplementary Fig. S1: KEGG analysis of up-regulated genes (colored in orange) and down-regulated genes (colored in green).

Supplementary Fig. S2:

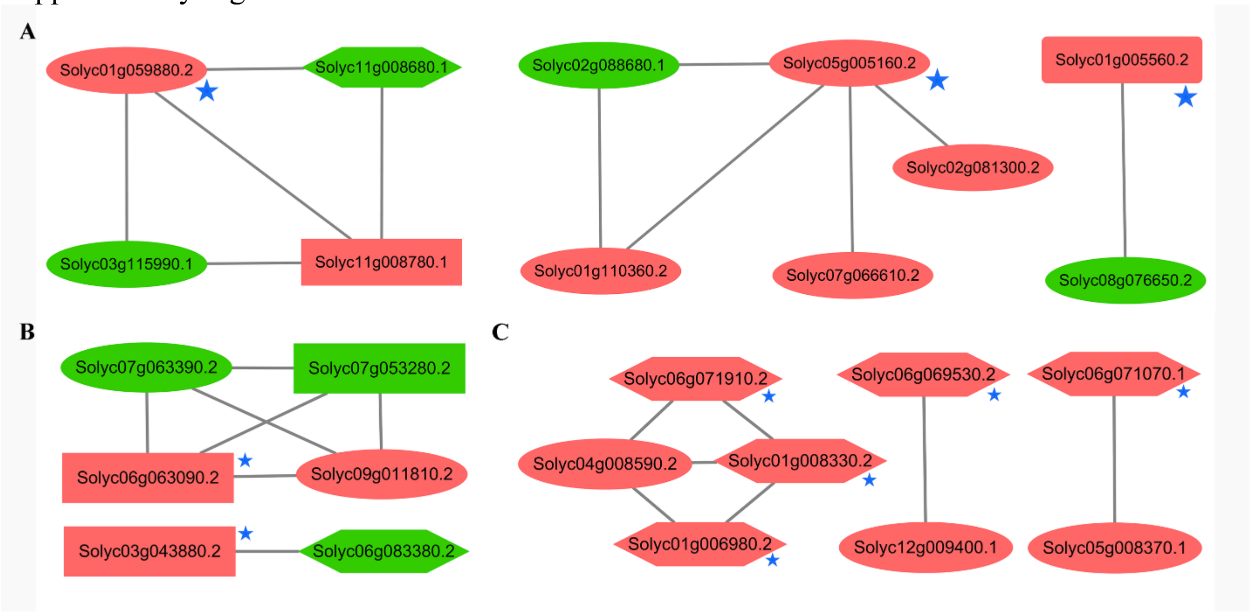

Supplementary Fig. S2: Co-expression analysis of genes involved in sugar and organic acid (A), amino acid (B) as well as fatty acid (C) schematic pathway. Up-regulated genes were colored in red while down-regulated genes were colored in green.

Supplementary Fig. S3: Quality control of RNA-seq data

RNA-seq Base Control (Low Temperature, Day 7)

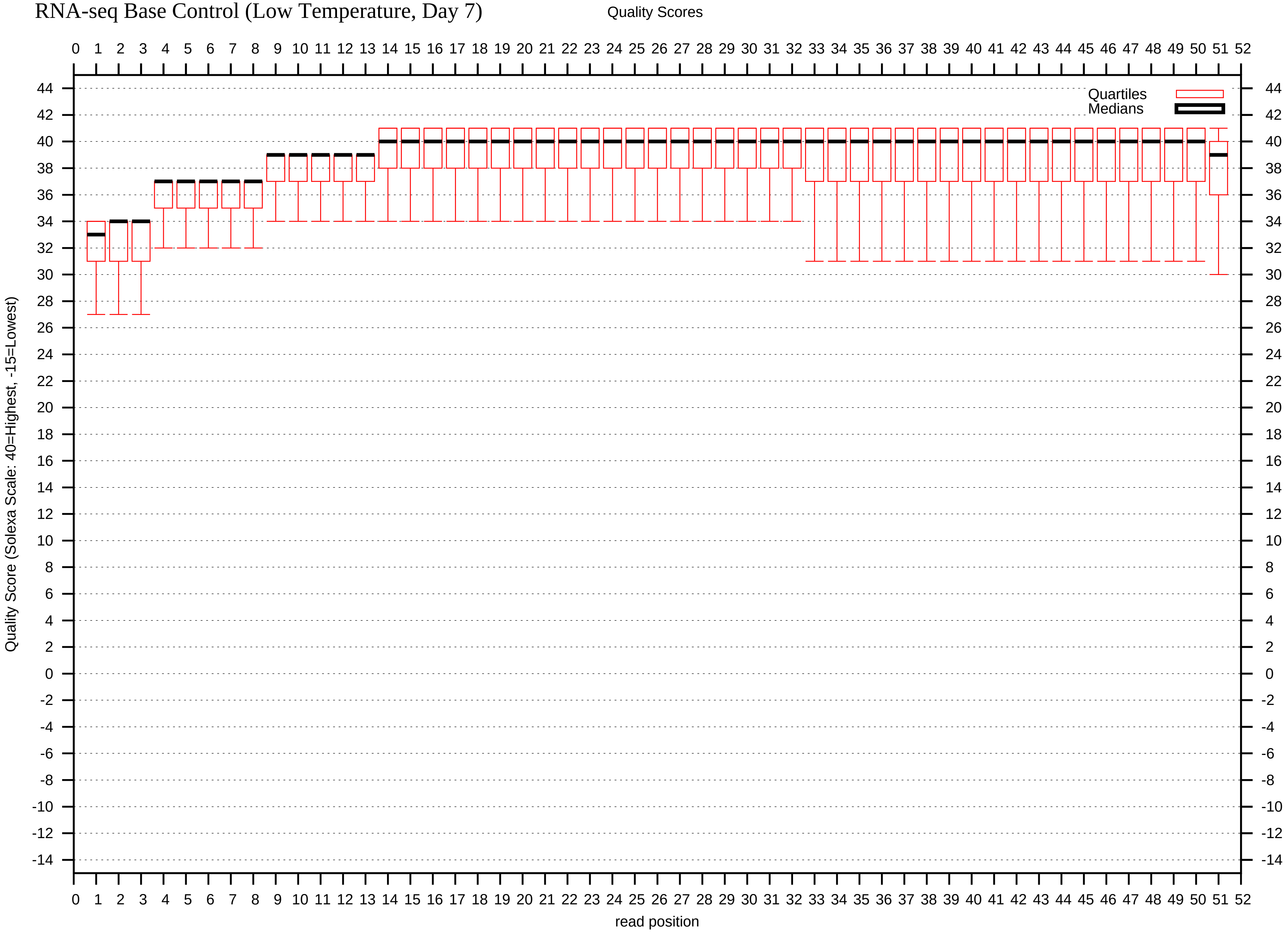

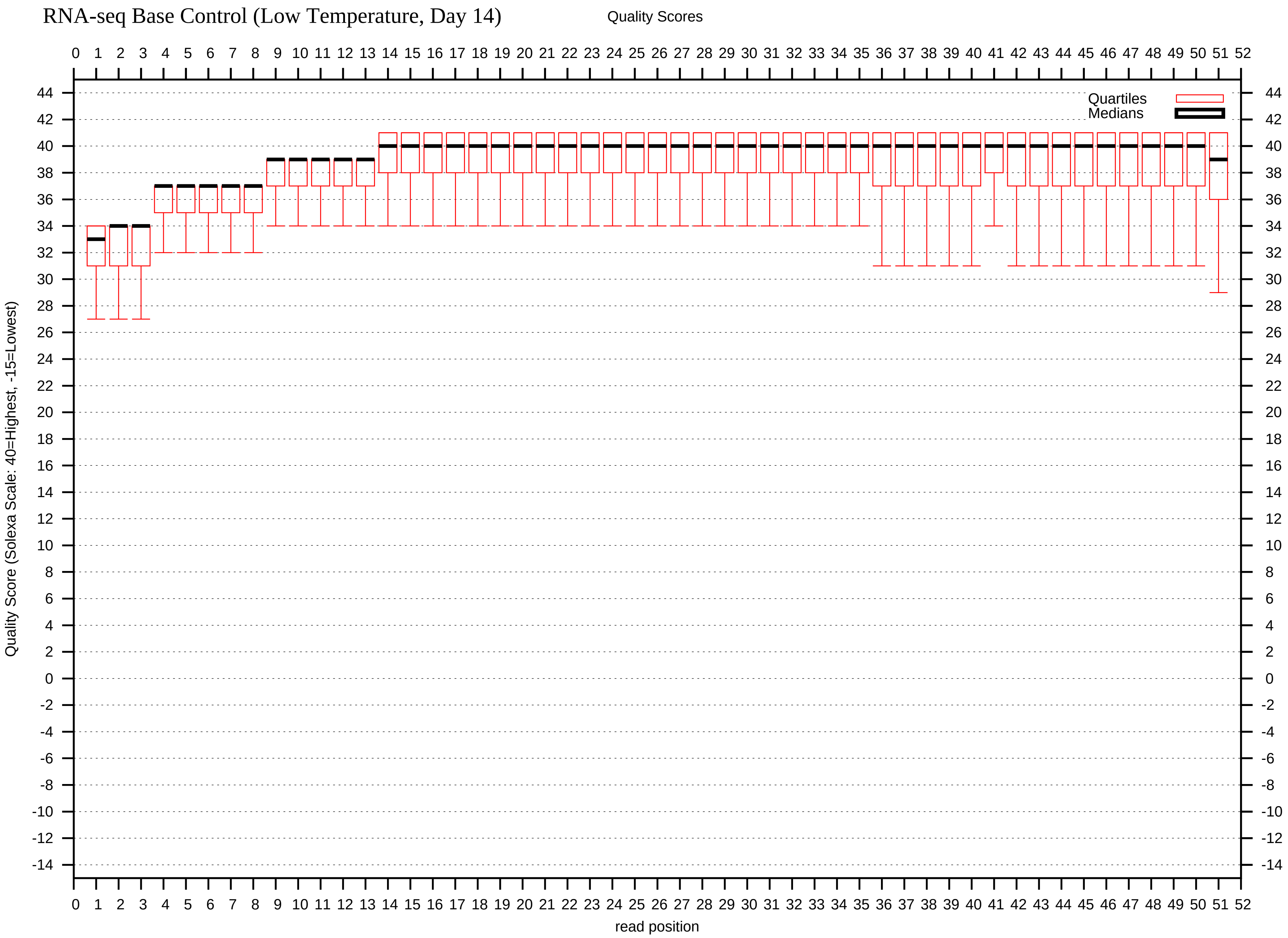
RNA-seq Base Control (Low Temperature, Day 14)
Quality Scores
Quartiles
Medians
Quality Score (Solexa Scale: 40=Highest, -15=Lowest)
read position

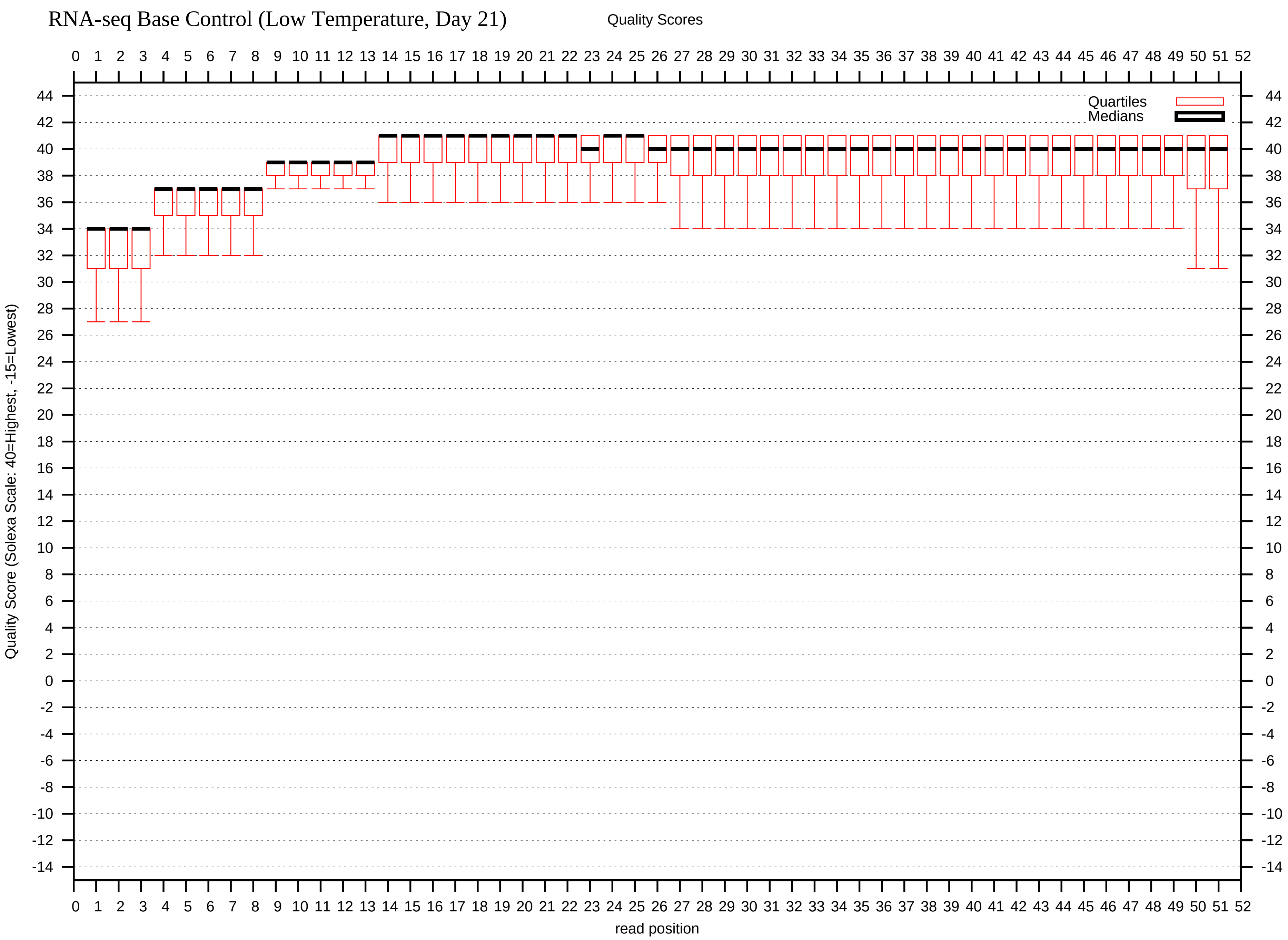
RNA-seq Base Control (Low Temperature, Day 21)
Quality Scores
Quartiles
Medians
Quality Score (Solexa Scale: 40=Highest, -15=Lowest)
read position

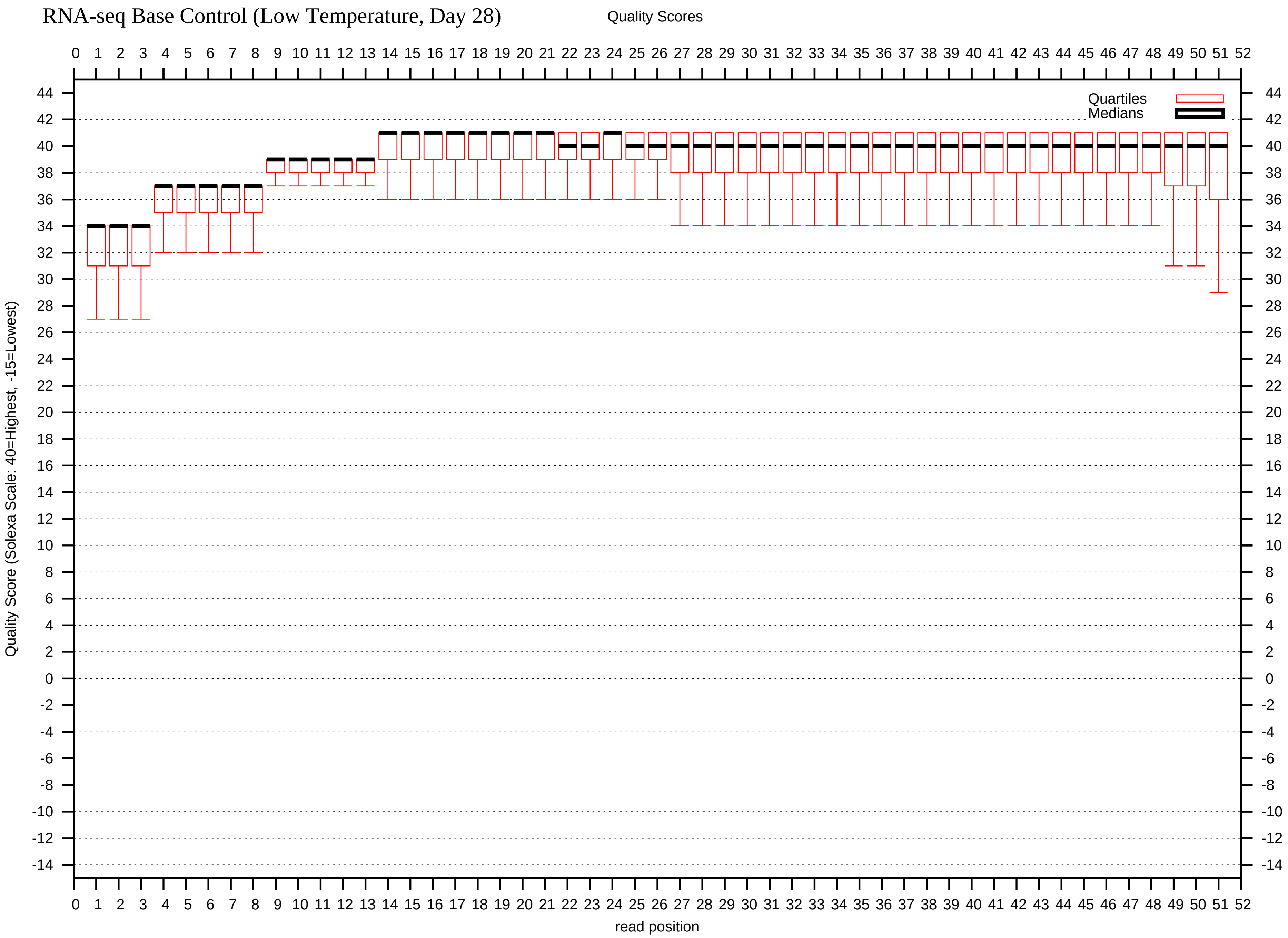

RNA-seq Base Control (Low Temperature, Day 28)
Quality Scores
Quartiles
Medians
Quality Score (Solexa Scale: 40=Highest, -15=Lowest)
read position

RNA-seq Base Control (Room Temperature, Day 7)

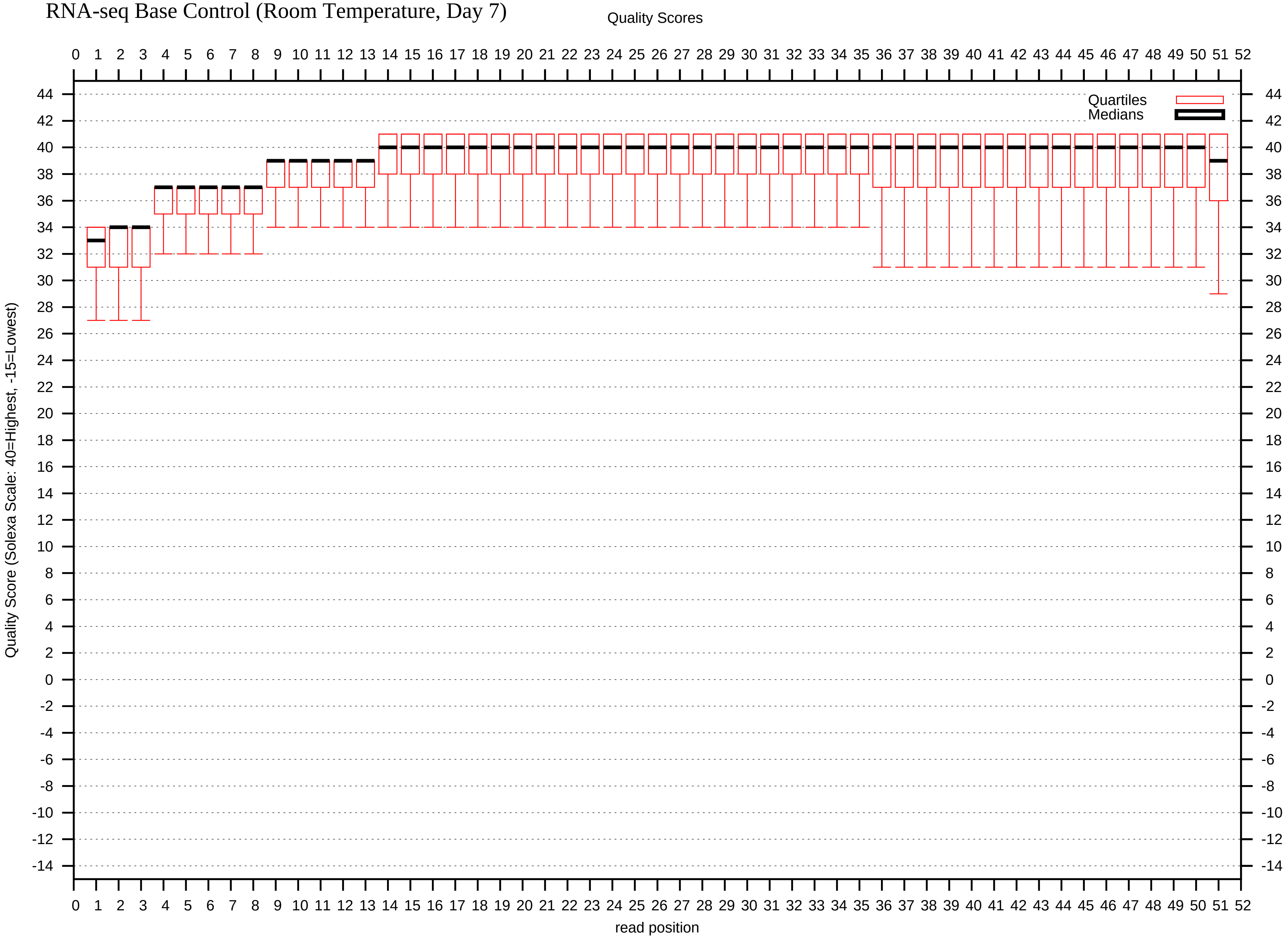

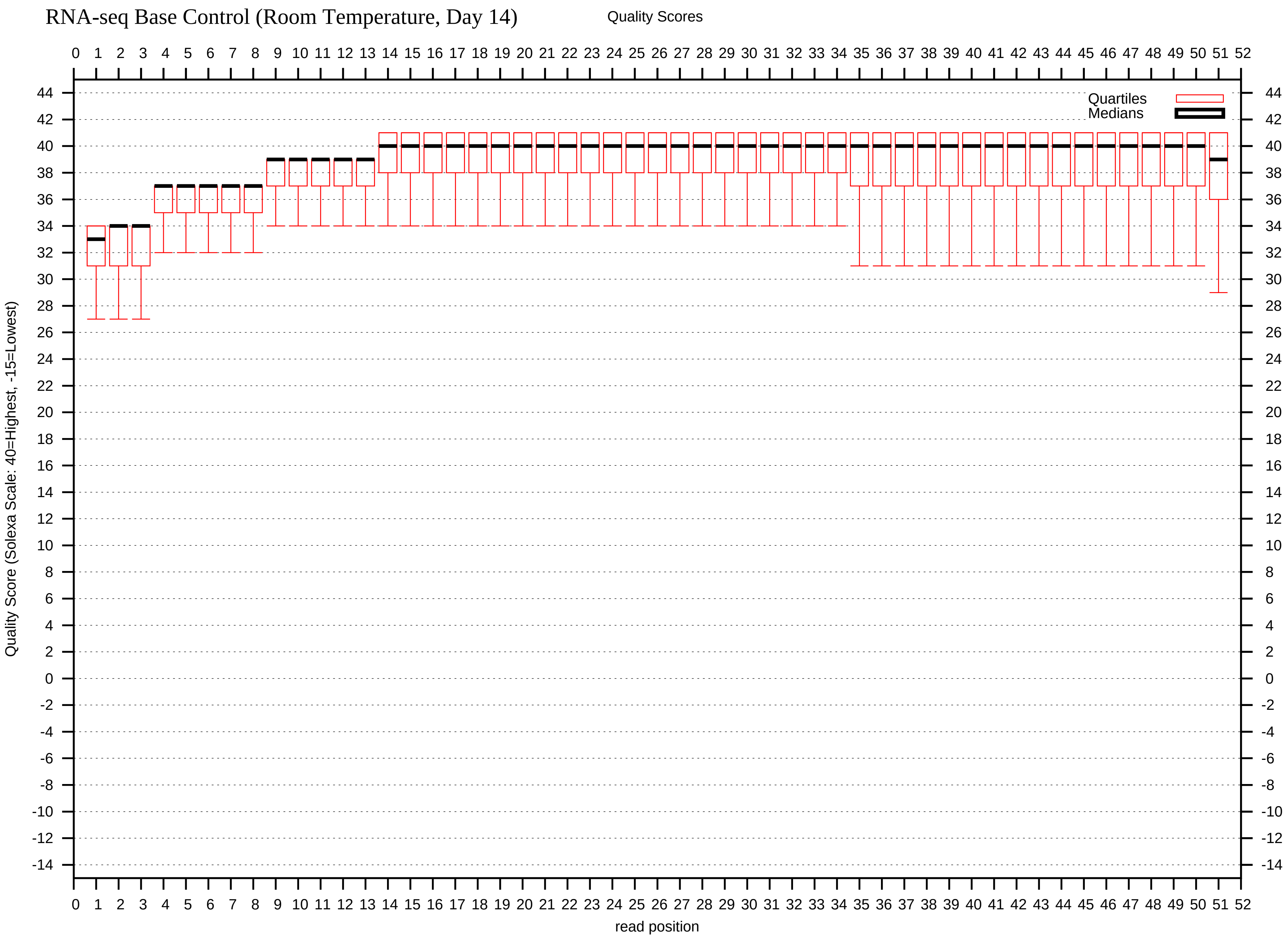
RNA-seq Base Control (Room Temperature, Day 14)
Quality Scores
Quartiles
Medians
Quality Score (Solexa Scale: 40=Highest, -15=Lowest)
read position

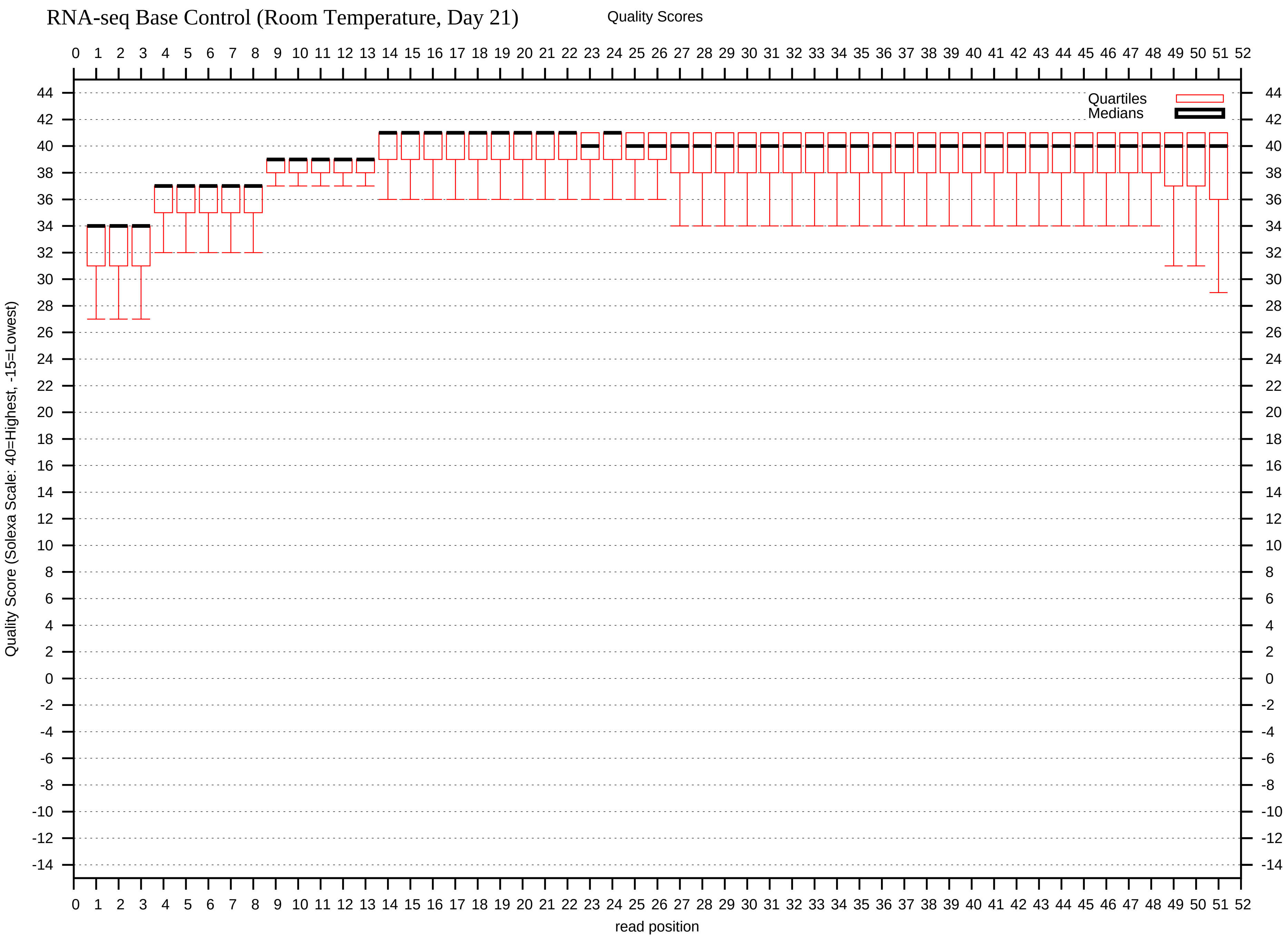
RNA-seq Base Control (Room Temperature, Day 21)
Quality Scores
Quartiles
Medians
Quality Score (Solexa Scale: 40=Highest, -15=Lowest)
read position

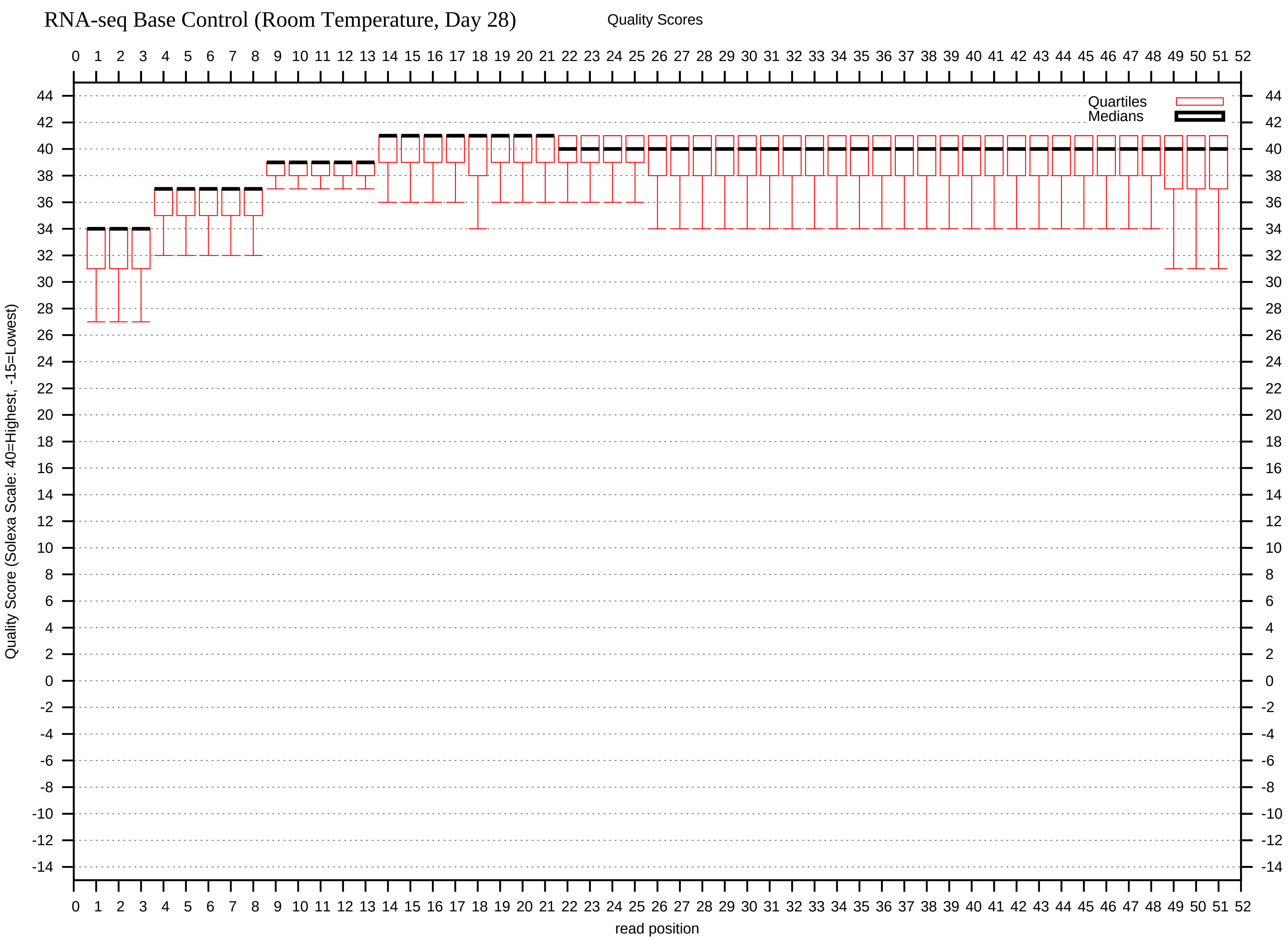

RNA-seq Base Control (Room Temperature, Day 28)
Quality Scores
Quartiles
Medians
Quality Score (Solexa Scale: 40=Highest, -15=Lowest)
read position

RNA-seq reads quality of room temperature (RT) and low temperature (LT) on day 7, 14, 21 and 28. Raw data has been filtered to get clean data and Q20 correspond to 1 in 100 error probability.

Sample Name	Raw Reads Number	Raw Base number （bp）	≥ Q20(%)	Clean Reads Number	Clean Base Number （bp）	≥ Q20(%)
RT-7d	18181514	927257214	98.25	16705619	830158190	98.7
RT-14d	18681722	952767822	98.27	17142445	851827204	98.69
RT-21d	19558618	997489518	98.66	17920013	890635024	99.02
RT-28d	16323527	832499877	98.67	14992449	745662775	99.02
LT-7d	29983796	1529173596	98.19	27561428	1370110456	98.64
LT-14d	20394797	1040134647	98.3	18770644	933444094	98.7
LT-21d	18864564	962092764	98.66	17338724	862492901	99.05
LT-28d	15787151	805144701	98.63	14499691	720972504	99.03
